# Mucosal Microbiome Profiles Polygenic Irritable Bowel Syndrome in Mestizo Individuals

**DOI:** 10.3389/fcimb.2020.00072

**Published:** 2020-03-20

**Authors:** Rene Arredondo-Hernández, Max Schmulson, Patricia Orduña, Gamaliel López-Leal, Angel-Mario Zarate, Gerardo Alanis-Funes, Luis David Alcaraz, Rubí Santiago-Cruz, Miguel A. Cevallos, Antonio R. Villa, Samuel Ponce-de-León Rosales, Yolanda López-Vidal, Consorcio Mexicano para el Estudio del Microbioma Humano

**Affiliations:** ^1^Laboratorio de Microbioma, División de Estudios de Posgrado y División de Investigación, Facultad de Medicina, Universidad Nacional Autónoma de México, Mexico City, Mexico; ^2^Laboratorio de Hígado, Páncreas y Motilidad (HIPAM), Unidad de Investigación en Medicina Experimental, Facultad de Medicina, Universidad Nacional Autónoma de México, Mexico City, Mexico; ^3^Programa de Inmunología Molecular Microbiana, Departamento de Microbiología y Parasitología, Facultad de Medicina, Universidad Nacional Autónoma de México, Mexico City, Mexico; ^4^Hospital General de México, Dr. Eduardo Liceaga, Mexico City, Mexico; ^5^Tecnologico de Monterrey, School of Medicine and Health Sciences, Monterrey, Mexico; ^6^Departamento de Biología Celular, Facultad de Ciencias, Universidad Nacional Autónoma de México, Mexico City, Mexico; ^7^Centro de Ciencias Genómicas, Programa de Genómica Evolutiva, Universidad Nacional Autónoma de México, Cuernavaca, Mexico

**Keywords:** IBS, SNPs, microbiota, *Bacteroides*, polygenic SNPs

## Abstract

Irritable bowel syndrome (IBS) is the most frequent functional gastrointestinal disorder, worldwide, with a high prevalence among Mestizo Latin Americans. Because several inflammatory disorders appear to affect this population, a further understanding of host genomic background variants, in conjunction with colonic mucosa dysbiosis, is necessary to determine IBS physiopathology and the effects of environmental pressures. Using a simple polygenic model, host single nucleotide polymorphisms (SNPs) and the taxonomic compositions of microbiota were compared between IBS patients and healthy subjects. As proof of concept, five IBS-Rome III patients and five healthy controls (HCs) were systematically studied. The human and bacterial intestinal metagenome of each subject was taxonomically annotated and screened for previously annotated IBS, ulcerative colitis, and Crohn's disease-associated SNPs or taxon abundance. Dietary data and fecal markers were collected and associated with the intestinal microbiome. However, more than 1,000 variants were found, and at least 76 SNPs differentiated IBS patients from HCs, as did associations with 4 phyla and 10 bacterial genera. In this study, we found elements supporting a polygenic background, with frequent variants, among the Mestizo population, and the colonic mucosal enrichment of *Bacteroides, Alteromonas, Neisseria, Streptococcus*, and *Microbacterium*, may serve as a hallmark for IBS.

## Introduction

Irritable bowel syndrome (IBS) is a multifactorial disorder, caused by abnormalities within the gut-brain axis and resulting in autonomic hypersensitivity and gastrointestinal motility dysfunction (Distrutti et al., 2016). IBS is often accompanied by low-level inflammation, resulting from an increased interleukin (IL)18, IL1b, and myeloperoxidase effector production and activity (Aerssens et al., 2009; McKernan et al., 2011; Clarke et al., 2012). Although clinically divided into subtypes, evidence suggests that the genomic backgrounds that regulate neuro-immune traits and genomic differences among gut microbiota may underlie pathology and symptom display. Although gut microbiota varies throughout life, dysbiosis far exceeds the normal 10% variation range in IBS patients, with a decrease in butyrate producers, which favors the proliferation of Firmicutes and Proteobacteria phylum (Mayer et al., 2014). Currently, corticotropin-releasing factor (CRF), NOD-like receptor family pyrin domain containing 6 (NLRP6 or IDO1), nucleotide-binding oligomerization domain-containing protein 2 (NOD2), Toll-like receptor 4 (TLR4), and cytochrome P450 1A (CYP1a) have been associated with low butyrate levels and colonic mucosal inflammation in humans and animal model of IBS, demonstrating the effects of genomic background on cross-signaling (Vujkovic-Cvijin et al., 2015; Wang et al., 2016; Layunta et al., 2018; Manzella et al., 2018; Martin-Gallausiaux et al., 2018; Zhao et al., 2018; Yu et al., 2019). Although hypothesis-free analyses, such as genome-wide association studies (GWAS) may be able to disentangle all possible interactions, a major drawback of IBS standardized trials is that most have been low-powered and primarily focused on participants with Caucasian ancestry (Gazouli et al., 2016). Previous reports have shown gene variants that are frequently associated with Amerindian inheritance, such as hepatocyte nuclear factor 4 (HNF4)-Diabetes Mellitus 2, and ulcerative colitis-associated, may widely diverge from HapMap (Wang et al., 2016; Granados-Silvestre et al., 2017). However, shotgun metagenome sequencing and analysis remains a fast and sensitive alternative for the exploration of gene network variants and microbiota phylogeny among a standardized cohort, providing the opportunity to explore both the microbial community and host interactions in IBS patients with particular genetic backgrounds (Sharpton, 2014).

The current research aimed to contribute to a deeper understanding of gene variations and single nucleotide polymorphisms (SNPs) in a Mestizo validation cohort of IBS patients and variations in the gut microbiota, at the genus taxonomic level, in close contact with the colonic mucosal lining.

## Methods

### Study Population

Five IBS patients and 5 healthy controls (HCs) were recruited at the Hospital General de México Dr. Eduardo Liceaga, in Mexico City. This hospital is the largest general hospital of the Mexican public health system and serves patients that are referred from all over the country. Subjects of both sexes between 18 and 65 years old were included. All subjects completed the Rome III Adult Symptom Questionnaire. The IBS patients fulfilled the Rome III criteria and were further classified, according to their bowel habit subtypes, as IBS with diarrhea (IBS-D), IBS with constipation (IBS-C), mixed IBS (IBS-M), or unclassified IBS (IBS-U). HCs were recruited through invitations sent to the patients' relatives and advertisements. HCs did not meet the Rome III criteria for IBS or any other functional bowel disorder. Subjects who fulfilled the IBS criteria were also evaluated using the IBS severity scoring system (IBS-SSS) and were classified as mild (75 to <175), moderate (175–300), or severe (>300) (Francis et al., 1997; Almansa et al., 2011). The presence of organic gastrointestinal diseases, such as inflammatory bowel disease (IBD), celiac disease, and infectious gastroenteritis, or systemic diseases, such as diabetes, thyroid, autoimmune, or any coagulation disorders, was ruled out in all subjects. In addition, subjects were excluded if they received antibiotics or proton pump inhibitor treatment during the previous 3 months. All subjects underwent a complete colonoscopy, after bowel cleansing the evening before, using 4 sachets of polyethylene glycol/electrolytes dissolved in 4 liters of water (1 sachet/liter). Each sachet contained 105.0 g macrogol 3350, 1.43 g sodium bicarbonate, 2.80 g sodium chloride, and 0.37 g potassium chloride. Both the IBS patients and HCs reported to the Endoscopy Unit of the Hospital General de Mexico after an 8-h fasting period. After signing informed consent, they were given a colonoscopy under IV sedation. No patient had any colonoscopic abnormalities.

All subjects answered the Rome III Questionnaire and a Frequency of Food Intake Questionnaire, which was analyzed by Nutrients Analysis System (SNUT) software to determine the dietetic contents. Correlations between microbiota and dietary nutrients were conducted using Spearman's (Rho) and Pearson's (*r*) correlation coefficients, according to sample distribution. A *p* ≤ 0.05 was considered to be significant.

This study was approved by the Ethics and Research commissions of the Faculty of Medicine at Universidad Nacional Autónoma de México (UNAM) (124/2014) and the respective commissions at the Hospital General de México (DI/15/UME/03/33).

### Biopsy Sampling

Four biopsies were collected from the descending colon. The biopsies were placed into cryotubes, containing 500 μl RNAlater solution, and were transported at room temperature from the hospital to the Programa de Inmunologia Molecular Microbiana at the Faculty of Medicine at Universidad Nacional Autónoma de México (UNAM), where they were processed.

### Genomic DNA Extraction and Sequencing

DNA (mean concentration 4.46 μg) was extracted from colon biopsies with the QIAamp® DNA mini kit (Qiagen, No.51306), following the manufacturer's instructions. The DNA quality was determined by agarose gel electrophoresis, and purity and quantity were determined by measuring 260/280 absorbance ratios (NanoDrop, ThermoFisher, USA).

### Sequencing

DNA shotgun sequencing was performed on an Illumina HiSeq 4000 platform, at Macrogen Inc. (Seoul, Korea). Here, a TruSeq DNA PCR Free library (Illumina, USA) was constructed, and the sequencing was performed using a 2 × 100 bp paired-end format. Sequencing data were deposited at NCBI under the SRA accession number SUB6452398 and at BioProject ID PRJNA579180.

### Sequence Processing

(1) *Sequence quality*. The sequences obtained from each sample (164,935,740–233,353,722 bp) were analyzed with FastQC software (v. 0.1.1.5), and low-quality sequences (*Q* < 20) were detected and removed. (2) *Merged reads*. The forward and reverse reads for each sample were merged, using PEAR software (v. 0.9.6), using the default settings, to increase the total length. (3) *Sequence alignment against the human genome*. All the samples were mapped with Bowtie software (v. 1.2.0), using the default settings, to align the reads against the human genome (Homo sapiens, GRCh38). On average, the number of non-aligned sequences returned 19,763,708 reads, whereas aligned sequences returned 193,086,669 reads, which were eliminated from the analysis.

### Taxonomic Annotation

The sequences that were not aligned against the human genome were uploaded to the Metagenomics Rapid Annotation using Subsystems Technology server (MG-RAST), which compares the sequences against the non-redundant protein database M5nr (Keegan et al., 2016). We used the lowest common ancestor (LCA) method for taxonomic assignation, and mapped reads were considered based on the following parameters: an *e*-value 1e-5, minimum identity 60%, and 30-bp minimum alignment length. Metagenome annotations are available in MG-RAST, with the accession numbers 4683929.3, 4683933.3, 4683935.3, 4698475.3, 4700742.3, 4698476.3, 4702949.3, 4700744.3, and 4700745.3.

### Data Management

The abundant MG-RAST profiles were transformed into relative frequencies for easier data handling. The relative abundances were plotted using phyloseqGraphTest and ggplot2 packages in R (v. 3.4.3). Finally, we conducted a statistical analysis of the relative frequencies of the gut microbiome genera relative to the identified SNPs, using the Mann-Whitney *U*-test in SPSS software (v. 12).

### Genes and SNPs Associated With Gastrointestinal Disease

**Figure 1:**
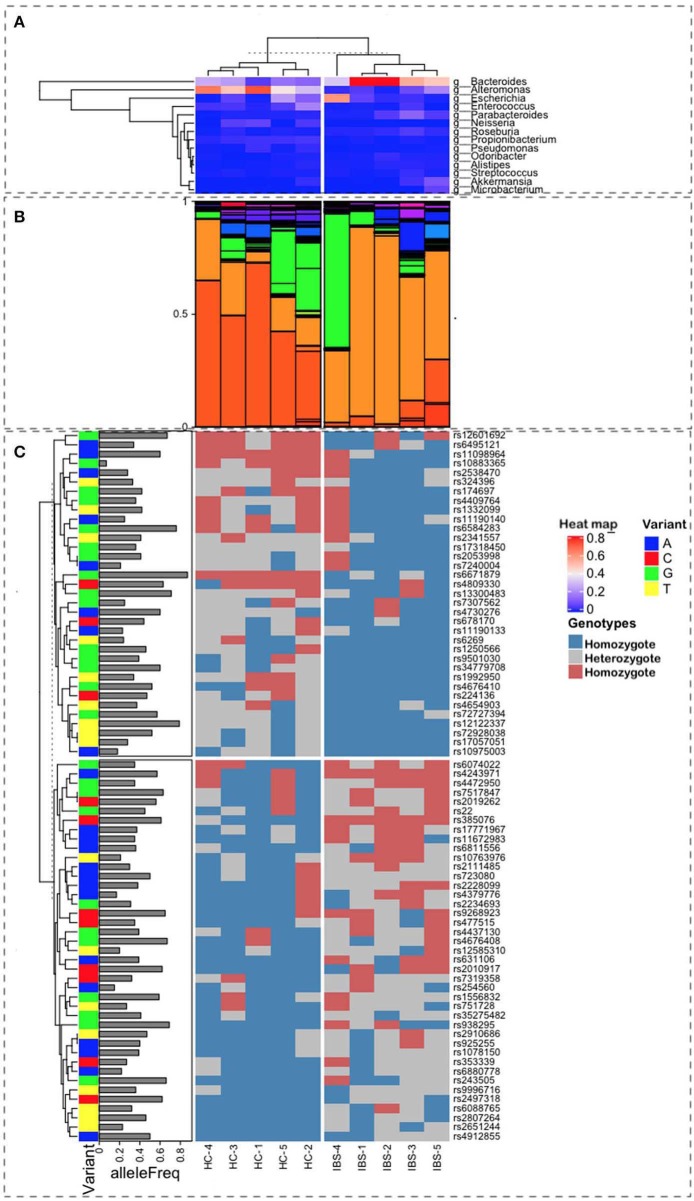
Representation of the multiple sources of information used for this study. Ten samples were characterized, 5 as Healthy Control (HC), on the left-side plots, and 5 as Irritable Bowel Syndrome (IBS), on the right-side plots, with labels at the bottom of the figure. **(A)** Heatmap from a hierarchical clustering algorithm showing the level of presence for the top microbes at the genus level in both groups. Bacteroides are the most frequent in IBS, whereas the Alteromonas population is higher in HC samples **(B)**. Relative proportions of microbial taxa at the genus level show a clear difference in patterns between HC and IBS (light and dark orange). **(C)** Correlation with SNP variants associated with IBS and their allele frequencies in the Mexican population.

Variant annotation was performed using the Genome Analysis Toolkit (GATK), according to the Best Practices Pipeline published by the BROAD Institute (McKenna et al., 2010; van der Auwera et al., 2013). For data pre-processing, FASTQ files from our shotgun data were converted to BAM formats (not aligned) and aligned with the human genome (GRCh version 38 patch 13) using BWA (Li and Durbin, 2010). PCR duplicates were removed from reads files. Search variants (SNPs) were made with Haplotype Caller (van der Auwera et al., 2013). The SNPs identified in the samples were screened against known SNPs associated with intestinal bowel diseases (909 SNPs) and IBS (25 SNPs), based on the GWAS catalog, which was consulted in March 2019 and May 2019, respectively. Moreover, after reviewing the literature (RV), we identified 709 additional SNPs. In total, we identified 1,643 SNPs. Additionally, we conducted a search for genes previously associated with IBS and IBD, analyzing a total of 1,144 genes. Searches and annotations of variant information available in public databases were performed using Funcotator (Genome Annotation Toolkit v. 4.1.1.0) (Buniello et al., 2019). The IDs of SNPs were added using bcftools (v. 1.9) and dbSNP v. 1.1.1.1 (v. 151) (Sherry et al., 2001; Li, 2011; Kuhlwilm and Boeckx, 2019). We compared the presence of all identified SNPs between IBS patients and HCs, the SNPs that appeared differentially in at least three subjects between these groups were selected for further analysis. The Figure 1 was created using R software V.3.6.0 with devtools package as well as ComplexHeatmap package (Gu et al., 2016), which allows the user to stack all levels of information onto one image. Tables with information about genes, SNPs, microbiome, genotypes, and disease were uploaded into the R environment and code was developed to arrange the information as seen on the Figure 1.

## Results and Discussion

### Characteristics of the Study Subjects

The demographic and clinical characteristics of the population are shown in Table 1. IBS patients included 4 women and 1 man (mean age: 41 years), 3 who were classified as IBS-D, 1 as IBS-C, and 1 as IBS-M. HCs included 3 women and 2 men (mean age: 28 years).

**Table 1:** Demographic and clinical characteristics of the study population.

**Sample**	**Sex**	**Age**	**Marital status**	**Educational level**	**BMI**	**Comorbidities**	**IBS subtype**	**Severity**
IBS-1	W	41	Single	Middle school	Normal	None	IBS-C	Mild
IBS-2	W	60	Single	Technical	OW	None	IBS-D	Moderate
IBS-3	W	60	Widowed	Elementary	Normal	Hyperlipidemia	IBS-D	Moderate
IBS-4	W	37	Single	High school	Normal	None	IBS-D	Mild
IBS-5	M	24	Single	Higher degree	OW	None	IBS-M	Moderate
HC-1	M	24	Single	Higher degree	Normal	None	–	–
HC-2	W	33	Single	High school	Normal	None	–	–
HC-3	W	28	Single	Higher degree	Normal	None	–	–
HC-4	M	27	Single	Higher degree	OW	None	–	–
HC-5	W	44	Married	High school	OW	None	–	–

*IBS, irritable bowel syndrome; C, constipation; D, diarrhea; M, mixed; HC, healthy controls; W, woman; M, man; OW, overweight*.

### Metagenomic Results

Among the taxonomic annotations of sequences not aligned with the human genome, sequences were classified as belonging to Archaea, Bacteria, Eukaryotes, and Viruses. A lower percentage of bacterial sequences was found in IBS patients (average 45.06%) compared with HCs (average 90.55%) (Table S1).

The most representative phyla were Actinobacteria, Bacteroidetes, Firmicutes, Fusobacteria, Proteobacteria, and Verrucomicrobia, which were present in all metagenomes, with the exception of Fusobacteria, which was absent in one IBS patient (Table 2). Both Actinobacteria and Proteobacteria were found at higher frequencies in IBS patients compared with HCs. In contrast, the most representative phylum among HCs was Bacteroidetes. Significant increases in the Actinobacteria phyla (*p* = 0.028) and Proteobacteria (*p* = 0.009) and a significant decrease in Bacteroidetes (*p* = 0.009) was found in the IBS group compared with the HC group (Table 2). The genera found at higher relative frequencies in the metagenomes were *Bacteroides, Alteromonas, Neisseria, Streptococcus, Microbacterium*, and other previously described genera (Figure 1A). However, only *Bacteroides* was found in the metagenomes of all 10 subjects, with a higher relative frequency in HCs than in IBS patients. In the IBS group, significant increases in the relative frequencies of *Propionibacterium* (*p* = 0.009) and *Staphylococcus* (*p* = 0.047) and an increasing trend in the relative frequency of *Enterococcus* (*p* = 0.076) were observed compared with the HC group. In contrast, among HCs, *Dorea* (*p* = 0.016), *Faecalibacterium* (*p* = 0.047), and *Gemella* (*p* = 0.041) were significantly decreased compared with the IBS group (Figure 1B). We found no significant difference in Bifidobacterium between the IBS and HC groups. Interestingly, we found a higher frequency of the genus *Pseudomonas* among our IBS patients, which was determined by an increase only in the IBS-C and IBS-M patients, but not in the IBS-D patient.

**Table 2:** Relative abundance per bacterial phylum in colonic biopsies.

**Phylum**	**Relative abundance (****%****)**										
	**IBS**					**HC**					
	**1**	**2**	**3**	**4**	**5**	**1**	**2**	**3**	**4**	**5**	***p-value***
*Actinobacteria*	9.0	2.9	2.9	1.8	4.6	1.6	1.5	0.8	0.47	0.49	0.028
*Bacteroidetes*	24.9	22.1	34.6	64.4	32.9	50.8	22.5	72.6	56.3	54.3	0.009
*Firmicutes*	23.4	27.5	42.3	28.2	44.3	35.4	25.3	23.0	38.3	42.1	0.917
*Fusobacteria*	0	20.4	0.16	0.31	0.10	0.13	0.70	0.01	0.04	0.09	0.347
*Proteobacteria*	42.4	26.6	19.8	5.18	17.4	11.9	22.9	3.33	4.40	0.18	0.009
*Verrucomicrobia*	0.18	0.27	0	0	0.41	0	0	0	0.39	1.21	0.914

*IBS, irritable bowel syndrome; HC, healthy controls*.

### The Polygenic Presence in IBS Patients, Based on SNPs

In animal models, the host genomic background has been demonstrated to be more important to the composition of gut microbiota than the microbiota found at points of contact with other microorganisms. In humans, GWAS analyses have identified relationships between host SNPs and gut microbiota. The relevant metabolic pathways include those associated with tryptophan metabolism, which are synthesized by microbiota components that regulate intestinal homeostasis. In our study, the combination of SNPs in the 5 IBS patients, based on allele frequency, was calculated for the Mexican Mestizo population. We selected 76 SNPs that showed differences (presence/absence) between IBS and HC groups. Of these, 40 SNPs were associated with the susceptibility for IBS. The hierarchical ordination showed two groups, consisting of high- and low-frequency SNPs among Mestizos (Figure 1, Table S2). Even those SNPs with the lowest frequency of 0.2 should be capable of having an effect. Although we only examined a subsample of SNPs, because these SNPs were shared among subjects, they demonstrate the different genomic backgrounds between HCs and IBS patients. The SNPs in IBS were associated, at the phylum level, with Firmicutes, Proteobacteria, and Actinobacteria, suggesting that the observed increase in Proteobacteria may be due to aerobic metabolic inflammation. Other studies, including ours, have identified SNPs in the tryptophan metabolic pathway that affect IgA levels, intestinal barrier formation, antimicrobial peptide production, and other immune activities due to dysbiosis resulting in decreased levels of aryl hydrocarbon receptor (*AHR*) ligands. In our IBS group, we identified the following SNPs: rs6495121, which is found in the *AHR*, which regulates toxicity via cytochrome *P450 1A1* (*CYP1A1*) and G-protein-coupled receptor 35 (*GPR35*); rs2228099, a candidate for type II diabetes and prediabetes intermediate traits (Figure 1C and Table S1). We found 7 SNPs, with frequency values of 0.2 alleles among the Mestizo population, including 4 that were associated with IBS risk factors, rs10763976, rs4379776, rs12585310, and rs254560; and 3 were associated with IBS protection, rs10883365, rs7240004, and rs10975003 (Table 1).

### The SNPs Associated With the Central Nervous System in Irritable Bowel Syndrome (IBS)

The identified SNPs were integrated with what was already known about functional IBS diseases associated with the central nervous system (CNS) (Table S1). After the brain, the intestine is the second-most enervated organ in the body. The rs254560_AG SNP in the C5orf66 gene on chromosome 5 found in this study is similar to genes such as *CCND280, GLI381, or RB1CC182*, which have been described as regulatory CNS proteins that modify transcriptional activity and contribute to brain growth trajectory changes at different biological levels, ranging from cellular changes, brain organization differences and complex phenotypic traits (Kuhlwilm and Boeckx, 2019). The weights of common IBD risk alleles are significant determinants during chronic granulomatosis diseases (CGD) (Huang et al., 2016). Based on general linear models, gray matter (GM) alterations caused by SNPs and Early Adverse Life events (EALs) are predictive of IBS. These studies showed that catecholaminergic SNPs may interact with other genes and environmental factors, such as EALs, catechol-O-methyltransferase (*COMT*), and alpha-1D adrenergic receptors (*ADRA1D*) rs1556832 (Orand et al., 2015). In our study, we found that rs1556832 was heterozygous in IBS patients with mild and moderate symptoms. However, according to Orand et al. (2015), homozygous rs1556832 was associated with increases in the volume of somatosensory regions and the left precentral gyrus. In contrast, rs1556832 was significantly associated with GI symptom severity.

The GI tract contains approximately 95% of the total body serotonin content, which is also known as 5-hydroxytryptamine (5-HT) and is primarily synthesized by enteric endocrine cells (EECs). Enterochromaffin (EC) cells are the best-characterized subset of EECs and are the primary source of 5-HT in the gut (Kwon et al., 2019). EC cells are dispersed among epithelial cells in the mucosal layer of the GI tract and release 5-HT apically into the gut lumen and basolateral, in response to various mechanical and chemical stimuli. EC cells synthesize 5-HT from its precursor, L-tryptophan. Tryptophan hydroxylase (Tph) catalyzes the synthesis of 5-HT. Two isoforms of Tph enzymes regulate 5-HT synthesis, including Tph1, which is primarily found in EC cells, and dysregulated 5-HT signaling has been observed in patients with IBS. Because of the close proximity of gut microbiota and 5-HT producing EC cells in the gut mucosal layer, crosstalk between them is likely to play a critical role in the maintenance of intestinal homeostasis, and recently, gut bacteria have been shown to stimulate the release of 5-HT from EC cells. Two major bacterial phyla, Firmicutes and Bacteroidetes, and 5 minor bacterial phyla, Proteobacteria, Actinobacteria, Fusobacteria, Cyanobacteria, and Verrucomicrobia, comprise the gut microbiota in adult humans. According to *in vitro* studies, 5-HT affects the growth of *B. thetaiotaomicron* and *E. faecalis*, providing further support that 5-HT may be able to inhibit peroxisome proliferator-activated receptor (PPAR)-γ in a microbiota-dependent manner. Altogether, these findings suggest that 5-HT released from EC cells directly and indirectly (via modulation of b-defensin production) plays a crucial role in the regulation of the gut microbial composition.

Meanwhile, the *SLC2A9* gene was associated with an unclassified *Porphyromonadaceae* (for us only rs62295801) was identified, and these loci were associated with bacterial abundance. 5-HT strongly inhibits NOD2 expression, which may play a role in intestinal pathophysiology not only through its inherent innate immune role but also through interactions with other receptors, such as TLR2, and the modulation of the intestinal serotonergic system, by decreasing serotonin transporter (SERT) activity and expression (Layunta et al., 2018). Analysis of SERT activity showed NOD2 activation, suggesting that long-term NOD2 activation may decrease SERT expression via transcriptional and/or post-transcriptional mechanisms, offering an explanation for the observed reduction in 5-HT uptake (Layunta et al., 2018).

### The Interaction Between the Immune Response and the Microbiome in IBS

The multidimensional relationship among key factors involved in vitamin D receptor (VDR) signaling (bile acids and ω6 fatty acids in particular) and the gut microbiota have been supported by genetic associations between functionally related loci, such as the HF4A rs9996716_AG SNP in the SHROOM3 gene on chromosome 4. The Proopiomelanocortin (POMC), shroom family member 3 (SHROOM3) *Marinilabiliaceae* family rs9996716_GA 5.58 × 10–9–0.690 has previously been demonstrated to increase the degradation of tryptophan along this immune responsive pathway during IBS and also found in our study (Wang et al., 2016). However, the relationship between TLR activation and kynurenine pathway activity in IBS remains unknown. A differential downstream profile of kynurenine production, subsequent to TLR activation, was found in IBS patients compared with healthy controls (Clarke et al., 2012). This profile included observed alterations in TLR1/2, TLR2, TLR3, TLR5, TLR7, and TLR8. An exaggerated response to TLR8 agonist by all cytokines investigated was observed in IBS patients. In addition, enhanced TLR2-induced tumor necrosis factor (TNF)- α release, TLR3-induced IL-8 release, TLR4-induced IL1b and TNF-α release, TLR5-induced IL1b and TNF-α release, and TLR7-induced IL-8 release were elevated in IBS-D patients (McKernan et al., 2011). The intestinal epithelial cells (IECs) play a central role in the host-microbiota dialogue by inducing the first microbial-derived immune signals.

Among Firmicutes, the most active genera for indoleamine 2, 3-dioxygenase (IDO)-1 expression were *Clostridium, Lachnoclostridium, Ruminoclostridium*, and *Roseburia*, according to principal component analysis (PCA) and correlation analyses on short-chain fatty acid (SCFA) concentrations. *Clostridium butyricum* regulates the visceral hypersensitivity of IBS (Shon et al., 2015; Vujkovic-Cvijin et al., 2015; Martin-Gallausiaux et al., 2018). Approximately 40% of the colorectal biopsy specimens from IBS patients showed non-specific inflammatory manifestations, such as neutrophils, mast cells (MCs), and T cell infiltration, which is often referred to as low inflammation. Caspase-1, ASC, and CARD8 form NLRP, NF-κB, IL-1β, IL-18. *Clostridium butyricum* might play a beneficial role in visceral hypersensitivity of IBS by inhibiting low-grade inflammation of colonic mucous through its action on NLRP6 (Zhao et al., 2018). Polymorphisms in cytokine-encoding genes and alterations in allele and genotype frequencies have been reported in IBS patients and may result in changes in the production of some cytokines. SNPs in the IL-6 region have been associated with an increased risk of developing PI-IBS. These SNPs were identified as independent risk factors for developing PI-IBS (Lazaridis, 2018).

**Figure 2:**
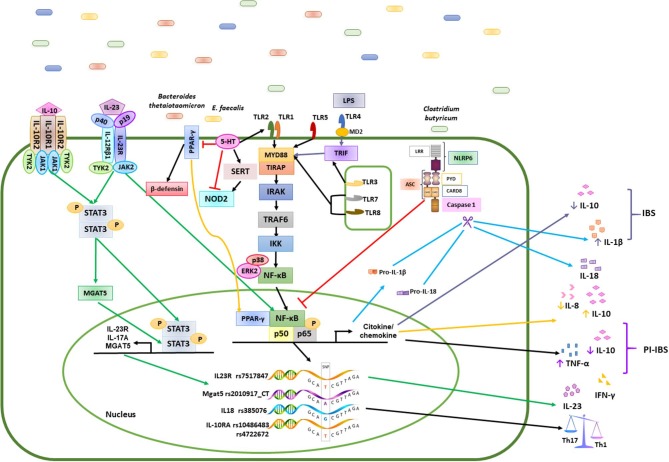
Mechanisms of the immune response induced by polygenic diseases related to the composition of the intestinal microbiota in patients with IBS and healthy subjects. Bacteria such as Bacteroidetes, Clostridium, *E. faecalis*, and Firmicutes, promote the activation of different signaling pathways in the epithelial cells of the intestinal mucosa through various mechanisms, while commensal bacteria stimulate the induction of a Th1/Th2 response by the production of cytokines such as IL-1β, IL-6, IL-10, IL-12, and TNF-α in healthy controls (HC). Differentiation of antigen-specific Th17 cells can be promoted by the increase in cytokine levels such as IL-1β, IL-8, IL-10, IL-17, IL-18, IL-23, TNF-α and INF-γ in patients with IBS. the green arrows represents the signaling pathway that is activated for the production of IL-23; the yellow arrows are the signaling pathway for the production of IL-8 and IL-10 in patients with IBS; the black arrows represents the path of activation of NFκB in healthy subjects; the blue arrows represent the caspase activation pathway and the production of IL1β and IL18; The truncated red line represents the inhibition of transcription factors or other molecules.

CXCL14 effectively promotes chemotaxis of immature, but not mature, DCs, both *in vivo* and *in vitro* (Tanegashima et al., 2010; Lu et al., 2016). Interestingly, CXCL14 shares striking common structural characteristics with them, including large, positively charged patches on its molecular surface, three anti-parallel β-strands, similar to those found in β-defensin, and a C-terminal α-helix that is typical for cathelicidin LL-37. *Enterococcus, Rosseburia, Propionibacterium, Streptococcus*, and *Microbacterium*, which were found in this study suggested that CXCL14 and human β-defensin described to have activity against Gram positive coccoid suggest an important question to be solved (Figure 2). Specifically, CXCL14 exhibits antimicrobial activity against Gram-negative *E. coli*, Gram-positive *Staphylococci* species, although a *Propionibacterium, Pseudomonas aeruginosa*, and *Streptococcus* species, which were found in this study needs to be described (Lu et al., 2016). The expression of *Cxcl14* in the CNS and the changes in neuropeptides, in addition to the metabolic changes described previously, suggested an important role for *Cxcl14* in the coordination of feeding behavior (Hernández-Ruiz and Zlotnik, 2017).

Because two SNPs (rs9268923_CT identified in this study, on chromosome 6 but not rs117782746) overlapped between CD and IBD although, the total number of unique identified SNPs was 64. Likewise, Nod2^−/−^ mice displayed higher expression levels of IFN-γ in their ileum but lacked signs of colitis and prolapse development. The scope and nature of gene-smoking interactions in IBD may be exemplary for other diseases. A focused analysis of the human leukocyte antigen (HLA) region revealed that only a subset of 16 IBD-predisposing alleles was found to interact with smoking. Several SNPs in the HLA region were also found to interact with smoking in relation to either CD, UC, or IBD (Yadav et al., 2017).

Binding to IL-23 receptor (IL-23R) complex encoded by the rs7517847 SNP gene on chromosome 1, was found in this study significantly increased the Janus kinase (JAK)-STAT and NF-κ B signaling pathways. IL-23/IL23R signaling plays critical roles in innate and adaptive inflammatory responses in the intestinal mucosa. As described previously, evidence indicated an association near the Smad7 gene, which encodes a protein that is overexpressed in IBD patients (Damas et al., 2017). This locus is relevant to IBS risk in the Hispanic population and may lead to a better understanding of the genetic architecture across this diverse population.

### Correlation Between Nutrients and Colonic Microbiome

Recently, we analyzed the correlation between nutrients and the microbiome and compared the results between IBS patients and HCs (Cano-Manrique et al., 2018). The results showed a higher intake of Fe (0.96 ± 0.1 vs. 0.61 ± 0.3 mg, *p* = 0.05) and vitamin B12 (5 ± 2.1 vs. 2.7 ± 0.7 mg, *p* = 0.04), suggesting a more animal-based diet in IBS patients. Significant correlations between CM and nutrients varied between the IBS and HC groups. In IBS, the phylum Proteobacteria correlated with Mn (*p* = 0.023) and monounsaturated fatty acids (MUFAs) (*p* = 0.03), possibly as a result of an animal-based diet, whereas in HCs, Proteobacteria correlated with lactose (*p* < 0.001). The genus *Ruminococcus*, a plant polysaccharide-utilizing bacterium, negatively correlated with cholesterol (*p* = 0.01) in IBS patients. *Faecalibacterium* correlated positively with cholesterol (*p* = 0.03) in IBS patients and negatively correlated with MUFAs in HCs (*p* = 0.03). *F. prausnitzii*, which was significantly decreased in IBS patients, was not correlated with any nutrients in IBS patients, whereas in HCs it was negatively correlated with CHO, glucose, and starch, possibly due to the fermentation of the starch during butyrate production.

## Conclusion

This study provides new evidence that 76 SNPs with differing penetrance might be involve in the susceptibility of IBS following a trend previously described for IBD. Since high frequency are often downgraded, the lowest 7 SNPs with allelic frequency among the Mestizo population could drive to dysbiosis. Of these, 2 SNPs were associated with IBD, 2 SNPs were associated with ulcerative colitis, 2 SNPs are intronic in the PARD 3 gene, associated with the regulation of serotonin, and the last SNP has previously been associated with IBS. We also found an enrichment of the Firmicutes, Proteobacteria, and Actinobacteria phyla, where enterobacteria, enterococcus, Akkermancia, Alteromonas, and others play important roles in the pathophysiology of polygenic IBS patients, associated with SNPs in TLRs, IL18, IL23, and IL17, which can acts as a pro or anti-inflammatory effect depending on environment. This enrichment may be associated with inflammatory regulatory activities, including reduced IL-10 levels and the inhibition of b-defensin production from colonic epithelial cells, which altogether results in the perpetuation of gut inflammation. In IBS, compared with controls, different correlations exist between SNPs, the colonic microbiome, and nutrients (Figure 2).

## Data Availability Statement

Sequencing data were deposited at NCBI under the SRA accession number SUB6452398 and at BioProject ID PRJNA579180.

## Ethics Statement

The studies involving human participants were reviewed and approved by Comisión de Investigación-ética, Facultad de Medicina, UNAM 125/2014. The patients/participants provided their written informed consent to participate in this study.

## Author Contributions

RA-H contributed to SNPs analysis and reviewed the manuscript. MS contributed to study concept, patient recruitment, grant support, and manuscript. PO performed pipeline for DNA extraction, contributed to the metagenomic analysis, and reviewed the manuscript. GL-L performed initial analyses and structured the analysis pipeline for metagenomes. RS-C performed DNA extraction and metagenomic analyses. A-MZ recruited patients and conducted colonoscopies and biopsies. LA contributed to analysis of pipelines for the metagenomes. GA-F contributed to the analysis and design of figures and reviewed the manuscript. AV performed statistical analyses. SP contributed to study concept, coordination, supervision, and reviewed the manuscript. YL-V contributed to study concept, coordination, supervision, analyses, grant support, and wrote and reviewed the manuscript. MC contributed to structure the analysis pipeline for the metagenomes.

### Conflict of Interest

SP is an Advisor for Becton-Dickinson and A-MZ is an advisor for Olympus. MS received grant support from Alfa-Sigma Mexico and Genova Diagnostics; advisory Board from Alfa-Sigma Mexico and Gemelli Biotech; consultant of Commonwealth Diagnostics International Inc.; speaker for Alfa-Sigma Mexico, Commonwealth Diagnostics International Inc., Mayoly-Spindler Mexico, and Takeda Mexico. The remaining authors declare that the research was conducted in the absence of any commercial or financial relationships that could be construed as a potential conflict of interest.

## Supplementary Material

Figure S1 Relative frequency of the genera in the analyzed metagenomes. Taxonomic differences at genus level in IBS and HC metagenomes. The most frequent genera in HC were *Bacteroides, Faecalibacterium, Ruminococcus*, and *Prevotella* to a lesser degree. In IBS patients, there is an increase in *Enterococcus, Bifidobacterium*, and *Acinetobacter* in sample IBS-1C.

Table S1 List of SNPs with differences between IBS and HC groups.

Table S2 Genes associated to SNPs of Table S1.
